# Selective portal vein occlusion with hepatic artery preservation reduces posthepatectomy liver failure: a retrospective cohort study

**DOI:** 10.1097/JS9.0000000000003548

**Published:** 2025-09-24

**Authors:** Jiming Ma, Shuo Jin, Jitao Wang, Liuqing Yang, Bingjun Tang, Canhong Xiang, Qiang Li, Pengfei Wang, Nan Jiang, Jianping Song, Yumei Li, Dongliang Yang, Yan Wen, Xuedong Wang, Jiahong Dong

**Affiliations:** a Hepatopancreatobiliary Center, Beijing Tsinghua Changgung Hospital, Key Laboratory of Digital Intelligence Hepatology, Ministry of Education, School of Clinical Medicine, Tsinghua Medicine, Tsinghua University, Beijing, China; b Research Unit of Precision Hepatobiliary Surgery Paradigm, Chinese Academy of Medical Sciences, Beijing, China; c Institute of Organ Transplantation and Bionics, Beijing Key Laboratory of Liver Transplantation and Bionic Manufacturing, Tsinghua University, Beijing, China; d Department of Information Administration, Beijing Tsinghua Changgung Hospital, Tsinghua University, Beijing, China; e Department of Hepatobiliary Surgery, The Second Qilu Hospital of Shandong University, Jinan, China; f Department of Otorhinolaryngology, Xiangyang No. 1 People’s Hospital, Xiangyang, China

**Keywords:** hepatectomy, hepatic inflow occlusion, posthepatectomy liver failure, propensity score matching, retrospective study, risk factors

## Abstract

**Background:**

Current hepatic inflow occlusion techniques have limitations in effectively preventing posthepatectomy liver failure (PHLF) from ischemia-reperfusion injury. Innovations in occlusion methods remain a critical area for advancement. This study investigated a hepatic inflow occlusion approach using selective portal vein occlusion (SPO) while maintaining hepatic arterial flow, aiming to evaluate its perioperative effects.

**Methods:**

Clinical data from consecutive patients who underwent hepatectomy between 2014 and 2024 were retrospectively collected. Postoperative outcomes were compared after a 1:1 ratio using propensity score matching (PSM) based on sex, age, body mass index, and Child–Pugh score using a fixed random seed. Univariate and multivariate logistic regression analyses were performed to identify risk factors for PHLF. Subgroup analyses were conducted to investigate the association between vascular occlusion strategies and the incidence of PHLF.

**Results:**

A total of 574 patients (192 SPO and 382 Pringle) were included. After PSM, 384 patients (192 SPO and 192 Pringle) were compared. PHLF was observed in 26 patients (6.8%). Hepatectomy with SPO was associated with a lower incidence of PHLF (3.1% vs. 10.4%, *P* = 0.026). No statistically significant difference was found in postoperative Clavien–Dindo grade III–IV complication rates between the two occlusion groups (7.3% vs. 13.0%, *P* = 0.165). The optimal cut-off value of ICG-R15 for predicting PHLF was identified as 6.9% based on receiver operating characteristic (ROC) analysis, with an area under the curve (AUC) of 0.830 (95% CI: 0.735–0.922), a sensitivity of 88.5%, and a specificity of 66.5%. In multivariate logistic regression analysis, blood loss (*P* = 0.019), ICG-R15 > 0.069 (*P* < 0.001), and undergoing >hemihepatectomy (*P* < 0.001) were identified as independent risk factors for PHLF. SPO was found to be an independent protective factor (*P* = 0.005). Subgroup analysis identified populations that benefit more from SPO, showing a significantly lower incidence of PHLF in patients aged <60 years (OR = 5.42, *P* = 0.019), males (OR = 5.06, *P* = 0.010), those with BMI ≥ 23 (OR = 3.81, *P* = 0.049), without cirrhosis (OR = 4.9, *P* = 0.003), with benign disease (OR = 5.07, *P* = 0.031), and undergoing ≤ hemihepatectomy (OR = 5.16, *P* = 0.005).

**Conclusion:**

The occlusion approach of SPO while preserving hepatic arterial flow can significantly reduce the incidence of PHLF.

**HIGHLIGHTS**

- SPO can effectively reduce the incidence of PHLF without increasing intraoperative blood loss or prolonging surgery time to an unacceptable extent.
- Compared with the Pringle maneuver, SPO significantly lowers the PHLF rate (3.1% vs. 10.4%, *P* = 0.026) without increasing the incidence of Clavien–Dindo grade III–IV complications (7.3% vs. 13.0%, *P* = 0.165) or other postoperative adverse events.
- SPO conferred greater benefit in specific patient subgroups, including those aged <60 years, without cirrhosis, with benign disease, and undergoing ≤hemihepatectomy.
- For patients those undergoing major hepatectomy (resection of more than a hemi-liver), SPO should be considered after excluding contraindications (e.g., anatomical variations of the hepatic hilum, tumor invasion of the hepatic hilum, or severe adhesions of the hepatic hilum).

## Introduction

The resection of hepatic segments and lobes has become the standard surgical approach for the treatment of both benign and malignant liver diseases, widely recognized in the field^[1,2]^. Since Lortat–Jacob reported the first modern right hepatectomy[3], liver resection techniques have seen significant development. With continuous advancements in surgical techniques, the perioperative morbidity and mortality rates of liver resection have decreased from approximately 20% in the early reports to less than 5% today^[4–6]^. However, perioperative mortality due to bleeding remains a major concern for surgeons. As a result, controlling intraoperative bleeding continues to be a key focus of research[7]. The complex vascular anatomy of the liver makes controlling and reducing bleeding particularly critical during liver resection to ensure the safety of the procedure. Effective hemorrhage control and minimizing blood transfusion not only influence the success of the surgery but also have long-term effects on the patients’ perioperative recovery and prognosis. Several studies have confirmed that significant intraoperative bleeding and transfusion are closely associated with poorer overall survival and recurrence-free survival^[8–11]^.

Bleeding control is primarily achieved through two key approaches: reducing the hepatic inflow by controlling the portal vein and hepatic artery, or minimizing venous reflux by regulating the hepatic venous outflow[12]. This process has evolved through several stages. In 1908, J.H. Pringle first introduced the complete hepatic inflow occlusion technique (Pringle maneuver), which has since become the most widely used method for controlling bleeding during liver resection[13]. The advantage of this technique lies in the fact that it does not require the dissection of the first hepatic portal, making it convenient and time-saving. However, this method also presents several drawbacks, including the risk of ischemia-reperfusion (I/R) injury when the occlusion is released, as well as limitations on the duration of the occlusion. The mechanism is likely closely related to the “cytokine storm” induced by inflammatory factors during reperfusion, as well as gut congestion, disruption of the intestinal barrier, and subsequent translocation of gut microbiota and endotoxins into the bloodstream^[14–16]^.

The unique anatomy of hepatic blood supply reveals that the liver typically receives approximately 25% of its blood supply from the hepatic artery and 75% from the portal vein. Importantly, the oxygen delivered by the hepatic artery accounts for 40–60% of the liver’s total oxygen demand^[17,18]^. In patients with liver cirrhosis, as fibrosis progresses, portal vein branches may become compressed or even occluded. In such cases, the hepatic artery undergoes compensatory changes, increasing the pressure gradient and enabling more efficient perfusion of the liver microvasculature. Studies have demonstrated that under these circumstances, the hepatic artery’s contribution to hepatic oxygen delivery can rise to as much as 75%. The unique dual blood supply of the liver makes selective portal vein occlusion (SPO) while preserving hepatic arterial flow a viable strategy to minimize bleeding and I/R injury during hepatectomy. However, this approach to reducing hepatic inflow has been rarely reported. Therefore, we conducted a retrospective study of patients undergoing hepatectomy using this method in our center to evaluate its clinical outcomes and safety.

## Methods

### Study design and participants

This retrospective cohort study covered the period from 2014 to 2024. Consecutive patients who underwent hepatectomy during this timeframe were included in the analysis. The exclusion criteria were as follows: lesions requiring skeletonization of the first hepatic hilum except for conducting main portal vein occlusion (e.g., vascular reconstruction, biliary-enteric anastomosis, lymphadenectomy); specific types of hepatectomy (e.g., *ex vivo* liver resection, caudate lobe resection); and cases with incomplete data (e.g., missing laboratory or imaging examination, or other critical information). Patient confidentiality and anonymity were rigorously maintained throughout the study, with all the data being anonymized prior to analysis. Informed consent was gained from every patient in the study; however, measures to protect patient data confidentiality were strictly upheld. Ethical approval for this study was obtained from the Clinical Research Ethics Committee of our institution (No. 24446-4-01). All procedures were conducted in accordance with the principles of the Declaration of Helsinki. This cohort study was reported in line with the Strengthening the Reporting of Cohort Studies in Surgery guidelines[19].

### Standard surgical procedures

Five consultant-level hepatobiliary surgeons, each with over 10 years of experience, performed the procedures to ensure consistency in technique and postoperative care. To reduce variability, a standardized surgical protocol was developed, and all surgeons were trained in both occlusion techniques and perioperative management.

And the choice of vascular occlusion strategy was guided by standardized criteria. Patients were excluded from SPO if preoperative evaluation revealed tumors involving the hepatic hilum or major hilar vessels, due to the complexity and higher risk associated with additional procedures such as vascular reconstruction or lymphadenectomy. Tumors located outside segments II–VIII, such as those invading the inferior vena cava or second hepatic hilum, were also excluded, as portal occlusion had limited effectiveness in controlling hemorrhage from these regions. For eligible patients, SPO was the preferred approach. However, if intraoperative findings (such as severe adhesions or unexpected vascular variations) made SPO unfeasible, the Pringle maneuver was used instead. The final occlusion strategy was determined based on both preoperative planning and intraoperative surgical judgment.

In the SPO group, skeletalization of the hepatoduodenal ligament was performed, involving the meticulous dissection of the hepatic artery, main portal vein, and bile duct. The main portal vein was clamped using a bulldog clamp to achieve complete blood flow occlusion (Supplemental Digital Content Figure S1, available at: http://links.lww.com/JS9/F198). After ligating the corresponding vessel supplied for the resected liver lobe or hemi-liver, the liver parenchyma was transected along the ischemic line. For veins and bile ducts with a diameter smaller than 2 mm at the transection plane, were selected to be coagulated by ultrasonic scalpel, cavitron ultrasonic surgical aspirator, or high-frequency electrosurgical equipment. For veins and ducts greater than 2 mm in diameter, ligation or titanium clips were applied for closure. The SPO method limited the duration of each occlusion cycle to no more than 45 minutes. If this time limit was exceeded, the occlusion was released for 5 minutes before reapplying.

In the Pringle group, hepatic blood flow was controlled using a tourniquet encircling the hepatoduodenal ligament. The tourniquet was gradually tightened until the pulsation of the hepatic artery was no longer touchable. The method of liver parenchyma transection was identical to that used in the SPO method. The occlusion time for this method was limited to a maximum of 15 minutes per cycle, followed by a 5-minute reperfusion period. Regardless of the occlusion method used, intraoperative ultrasound (particularly Doppler imaging) was routinely employed to objectively assess intrahepatic blood flow.

Complications, including posthepatectomy liver failure (PHLF), postoperation hemorrhage (POH), and bile leakage, were diagnosed according to the International Study Group of Liver Surgery (ISGLS) guidelines^[20–22]^. PHLF was defined as an elevated international normalized ratio (INR > 1.20) and hyperbilirubinemia (total bilirubin > 21.0 μmol/L) on or after postoperative day (POD) 5, based on the reference ranges used in our laboratory. PHLF was classified per ISGLS into three grades: Grade A required no management changes, Grade B required noninvasive treatment, and Grade C required invasive interventions (e.g., dialysis, ventilation, or liver support). Use of vasopressors due to circulatory failure was also considered Grade C. Other postoperative complications and 90-day mortality were evaluated using the Clavien–Dindo classification.

### Data collection

Baseline characteristics, including age; sex; body mass index (BMI); indocyanine green retention rate at 15 minutes (ICG-R15); American Society of Anesthesiologists physical status classification (ASA), cirrhosis, and Child–Pugh grade, were recorded. Intraoperative details included the surgery time, blood loss, number of ischemia episodes, ischemic duration, type of hepatectomy and occlusion, and lesion pathology. To assess postoperative laboratory changes, data including blood routine, liver and kidney function, and coagulation function levels were collected on POD 1–5. Other collected perioperative information included 90-day mortality, vascular intervention, re-operations, perioperative transfusion, intensive care unit (ICU) stay, and 30-day readmission.

### Statistical analysis

**Figure 1.**
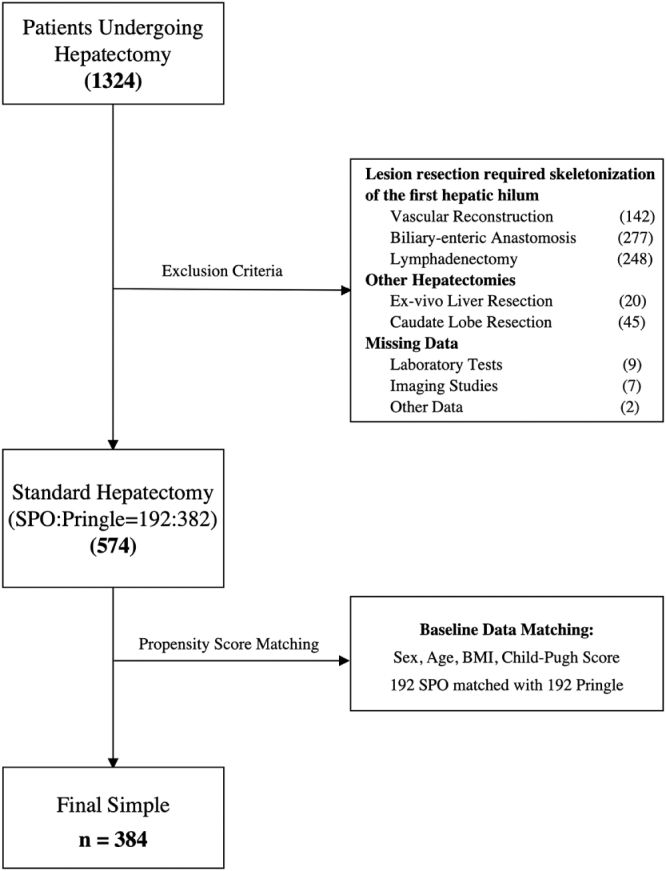
Flowchart: Method for sample selection, exclusion, matching, and determination of the final population.

SPO, selective portal vein occlusion; BMI, body mass index.

To mitigate selection bias and potential confounding factors, we performed 1:1 propensity score matching (PSM) based on sex, age, BMI, and Child–Pugh score. As a result, 192 patients were matched in each group using PSM (Fig. 1). Both groups consisted of patients from our center.

Numerical variables are expressed as medians (interquartile ranges, or IQRs) or as medians (± standard deviations, or SDs) if normally distributed. Categorical variables are expressed as numbers and proportions. The student’s *t*-test or Mann–Whitney *U* test was used to assess numerical variables, whereas Pearson’s χ² test was employed for categorical variables.

The predictive performance, sensitivity, and specificity of ICG-R15 for PHLF were assessed using ROC analysis, and the optimal cut-off value was determined based on the Youden Index.

Univariate and multivariate logistic regression models were used to assess the odds ratio (OR) and 95% confidence interval (CI) for the association between variables and PHLF incidence. The factors satisfying *P* value <0.05 in the univariate logistic regression analysis were included in the multivariate logistic regression analysis.

Subgroup analyses based on age, BMI, cirrhosis status, sex, pathology, and type of hepatectomy were performed to compare the incidence of PHLF between the Pringle maneuver and SPO. OR with 95% CI were calculated for each subgroup.

Statistical analyses were performed via Python (version 3.12.4) and R (version 4.4.2). *P* value <0.05 was considered significant.

## Results

### Clinical baseline characteristics and postoperative outcomes

During the study period, a total of 1324 patients underwent hepatectomy at our center. Among these, 750 patients met the exclusion criteria. Ultimately, after PSM, 384 patients were included in the study (Fig. 1).

During the preoperative data analysis, a total of 574 patients were involved. Prior to PSM, the Pringle group was associated with less blood loss (*P* = 0.001), shorter surgery time (*P* < 0.001), a higher proportion of segmental hepatectomy/ lobectomy (*P* = 0.002), and hepatocellular carcinoma (HCC) (*P* = 0.002). PSM with a 1:1 ratio resulted in 192 matched pairs for further analysis. In the matched cohort, both groups were well balanced in surgery time, blood loss, lesion pathology, type of hepatectomy, and other baseline characteristics, with all *P* values > 0.05. Among these patients, 167 (43.5%) were female, with a median age of 56 years. Cirrhosis was present in 53 patients (13.8%). Additionally, 133 patients (34.6%) were diagnosed with HCC, 64 (16.7%) with intrahepatic cholangiocarcinoma (ICC), 162 (42.2%) with benign disease, and 25 (6.5%) with other malignant diseases. Of these patients, 191 (49.7%) underwent hemihepatectomy, while 79 (20.6%) and 114 (29.7%) underwent segmental hepatectomy/lobectomy and extended hepatectomy/trisectionectomy, respectively. A total of 26 patients (6.8%) developed PHLF, while 15 patients (3.9%) underwent postoperative vascular intervention. Additionally, 45 patients (11.7%) went re-operation, and Clavien–Dindo Grade III–IV complications occurred in 39 patients (10.2%). The 90-day mortality rate was 2.6% (10 patients). For further details regarding other clinical aspects, please refer to Table 1.

**Table 1:** Baseline characteristics of the patients

Variables	Total (*n* = 384)	Before matching			After matching		
		Pringle (*n* = 382)	SPO (*n* = 192)	*P*	Pringle (*n* = 192)	SPO (*n* = 192)	*P*
Age (median [IQR]), years	56 (42–65)	56 (43–65)	56 (42–65)	0.801	56 (42–64)	56 (42–65)	0.906
Sex: Female (%)	167 (43.5%)	143 (37.4%)	85 (44.3%)	0.137	82 (42.7%)	85 (44.3%)	0.837
BMI (mean ± SD), kg/m^2^	22.48 ± 3.52	23.05 ± 3.73	22.43 ± 3.29	0.051	22.53 ± 3.74	22.43 ± 3.29	0.775
Cirrhosis (%)	53 (13.8%)	66 (17.3%)	27 (14.1%)	0.386	26 (13.5%)	27 (14.1%)	1.000
ICG–R15	0.05 (0.03–0.09)	0.06 (0.04–0.10)	0.05 (0.03–0.09)	0.092	0.06 (0.03–0.09)	0.05 (0.03–0.09)	0.421
Child–Pugh				0.046			0.554
Grade A (%)	331 (86.2%)	347 (90.8%)	163 (84.9%)		168 (87.5%)	163 (84.9%)	
Grade B (%)	53 (13.8%)	35 (9.2%)	29 (15.1%)		24 (12.5%)	29 (15.1%)	
Lesion pathology				0.002			0.274
HCC (%)	133 (34.6%)	170 (44.5%)	62 (32.3%)		71 (37.0%)	62 (32.3%)	
ICC (%)	64 (16.7%)	49 (12.8%)	38 (19.8%)		26 (13.5%)	38 (19.8%)	
Benign disease (%)	162 (42.2%)	126 (33.0%)	82 (42.7%)		80 (41.7%)	82 (42.7%)	
Other malignant disease (%)	25 (6.5%)	37 (9.7%)	10 (5.2%)		15 (7.8%)	10 (5.2%)	
ASA				0.200			1.000
I/II (%)	244 (63.5%)	220 (57.6%)	122 (63.5%)		122 (63.5%)	122 (63.5%)	
III/IV (%)	140 (36.5%)	162 (42.4%)	70 (36.5%)		70 (36.5%)	70 (36.5%)	
Type of hepatectomy				0.002			0.878
Segmental hepatectomy/Lobectomy	79 (20.6%)	131 (34.3%)	38 (19.8%)		41 (21.4%)	38 (19.8%)	
Hemihepatectomy	191 (49.7%)	154 (40.3%)	95 (49.5%)		96 (50.0%)	95 (49.5%)	
Extended hepatectomy/Trisectionectomy	114 (29.7%)	97 (25.4%)	59 (30.7%)		55 (28.6%)	59 (30.7%)	
Number of ischemia episodes (median [IQR]), times	3 (2–5)	3 (2–5)	3 (2–4)	0.498	4 (2–5)	3 (2–4)	0.071
Ischemic duration (median [IQR]), minutes	60 (40–95)	56 (38–89)	59 (42–91)	0.175	61 (38–98)	59 (42–91)	0.859
Intraoperative transfusion (%)	20 (5.2%)	20 (5.2%)	7 (3.6%)	0.522	13 (6.8%)	7 (3.6%)	0.251
Surgery time (median [IQR]), minutes	325 (247–418)	320 (226–350)	420 (276–490)	<0.001	312 (237–389)	374 (275–491)	0.293
Blood loss (median [IQR]), mL	300 (200–500)	300 (200–400)	300 (200–500)	0.001	300 (200–500)	300 (200–500)	0.607
PHLF	26 (6.8%)	36 (9.5%)	6 (3.1%)	0.038	20 (10.4%)	6 (3.1%)	0.026
Grade A (%)	4 (1.0%)	8 (2.1%)	0 (0.0%)		4 (2.1%)	0 (0.0%)	
Grade B (%)	13 (3.5%)	17 (4.5%)	4 (2.1%)		9 (4.7%)	4 (2.1%)	
Grade C (%)	9 (2.3%)	11 (2.9%)	2 (1.0%)		7 (3.6%)	2 (1.0%)	
Vascular intervention	15 (3.9%)	25 (6.5%)	4 (2.1%)	0.036	11 (5.7%)	4 (2.1%)	0.114
Re–operation	45 (11.7%)	44 (11.5%)	16 (8.3%)	0.302	29 (15.1%)	16 (8.3%)	0.057
Bile leakage (%)	67 (17.4%)	56 (14.6%)	29 (15.1%)	0.292	38 (19.8%)	29 (15.1%)	0.171
Grade A (%)	47 (12.2%)	39 (10.2%)	22 (11.5%)		25 (13.0%)	22 (11.5%)	
Grade B (%)	9 (2.3%)	5 (1.3%)	5 (2.6%)		4 (2.1%)	5 (2.6%)	
Grade C (%)	11 (2.9%)	12 (3.1%)	2 (1.0%)		9 (4.7%)	2 (1.0%)	
POH (%)	27 (7.0%)	30 (7.8%)	11 (5.7%)	0.054	16 (8.3%)	11 (5.7%)	0.168
Grade A (%)	12 (3.1%)	7 (1.8%)	7 (3.6%)		5 (2.6%)	7 (3.6%)	
Grade B (%)	7 (1.8%)	7 (1.8%)	3 (1.6%)		4 (2.1%)	3 (1.6%)	
Grade C (%)	8 (2.1%)	16 (4.2%)	1 (0.5%)		7 (3.6%)	1 (0.5%)	
Infection (%)	70 (18.2%)	72 (18.8%)	32 (16.7%)	0.210	38 (19.7%)	32 (16.7%)	0.304
Abdominal infection	29 (7.5%)	25 (6.5%)	14 (7.3%)		15 (7.8%)	14 (7.3%)	
Chest infection	25 (6.5%)	26 (6.8%)	13 (6.8%)		12 (6.2%)	13 (6.8%)	
Incisional infection	5 (1.3%)	3 (0.8%)	3 (1.6%)		2 (1.0%)	3 (1.6%)	
Infection of two or more sites	11 (2.9%)	18 (4.7%)	2 (1.0%)		9 (4.7%)	2 (1.0%)	
Clavien–Dindo				0.249			0.165
Grade I–II (%)	104 (27.1%)	87 (22.8%)	52 (27.1%)		52 (27.1%)	52 (27.1%)	
Grade III–IV (%)	39 (10.2%)	42 (11.0%)	14 (7.3%)		25 (13.0%)	14 (7.3%)	
Postoperative transfusion (%)	25 (6.5%)	27 (7.1%)	10 (5.2%)	0.499	15 (7.8%)	10 (5.2%)	0.408
Postoperative ICU stay (median [IQR]), days	1 (0–2)	1 (0–2)	0 (0–3)	0.404	1 (0–2)	0 (0–3)	0.386
The 30–day readmission (%)	31 (8.1%)	36 (9.4%)	14 (7.3%)	0.485	17 (8.9%)	14 (7.3%)	0.708
90–day mortality (%)	10 (2.6%)	12 (3.1%)	3 (1.6%)	0.400	7 (3.6%)	3 (1.6%)	0.336

ASA, American Society of Anesthesiologists physical status classification; BMI, body mass index; HCC, hepatocellular carcinoma; ICC, intrahepatic cholangiocarcinoma; ICG–R15, indocyanine green retention rate at 15 minutes; ICU, intensive care unit; IQR, interquartile range; PHLF, post–hepatectomy liver failure; POH, post–operation hemorrhage; SD, standard deviation; SPO, selective portal vein occlusion.

Among the 384 patients, there were no statistically significant differences in age (*P* = 0.906), sex (*P* = 0.837), Child-Pugh score (*P* = 0.554), type of hepatectomy (*P* = 0.878), number of ischemia episodes (*P* = 0.071), ischemic duration (*P* = 0.859), surgery time (*P* = 0.293), blood loss (*P* = 0.607), or other preoperative and intraoperative characteristics. But hepatectomy using the SPO was associated with a significantly lower incidence of PHLF (3.1% vs. 10.4%, *P* = 0.026). However, there were no statistically significant differences in other complications, including POH (5.7% vs. 8.3%, *P* = 0.168), bile leakage (15.1% vs. 19.8%, *P* = 0.171), infection (16.7% vs. 19.7%, *P* = 0.304), Clavien-Dindo grade III–IV complications (7.3% vs. 13.0%, *P* = 0.165), 90-day mortality (1.6% vs. 3.6%, *P* = 0.336), or other postoperative events.

**Figure 2.**
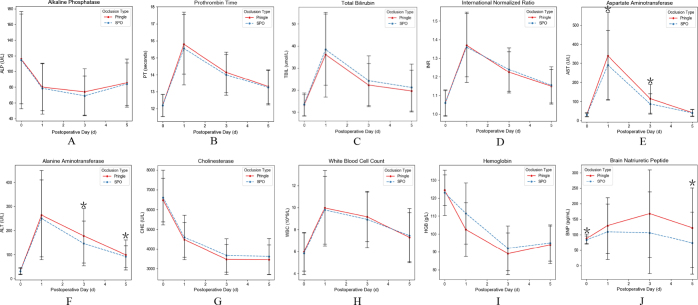
Comparison of laboratory tests between different types of occlusions after 1:1 propensity score matching.

As shown in Figure 2 and Supplemental Digital Content Table S1, available at: http://links.lww.com/JS9/F199, on POD 5, laboratory examination such as brain natriuretic peptide (BNP) and alanine aminotransferase (ALT) were significantly lower in the SPO group (median: 72.77 vs. 122.55 U/L, 91.65 vs. 98.90 U/L, *P* = 0.035 and *P* = 0.048, respectively). On POD 3, aspartate aminotransferase (AST) and ALT levels were also significantly lower in the SPO group (median: 87.35 vs. 114.90 U/L, 146.35 vs. 177.75 U/L, *P* = 0.022 and *P* = 0.040, respectively). On POD 1, AST levels were significantly lower in the SPO group (median: 291.15 vs. 339.30 U/L, *P* = 0.042).

Comparison of (A) alkaline phosphatase, (B) prothrombin time, (C) total bilirubin (D) international normalized ratio, (E) aspartate aminotransferase, (F) alanine aminotransferas, (G) cholinesterase, (H) white blood cell count, (I) hemoglobin, (J) brain natriuretic peptide in patients in selective portal vein occlusion and Pringle maneuver groups. Serial measurements in A–I are presented as mean. ^*^P  <  0.05.

### The predictive value of ICG-R15 for PHLF

**Figure 3.**
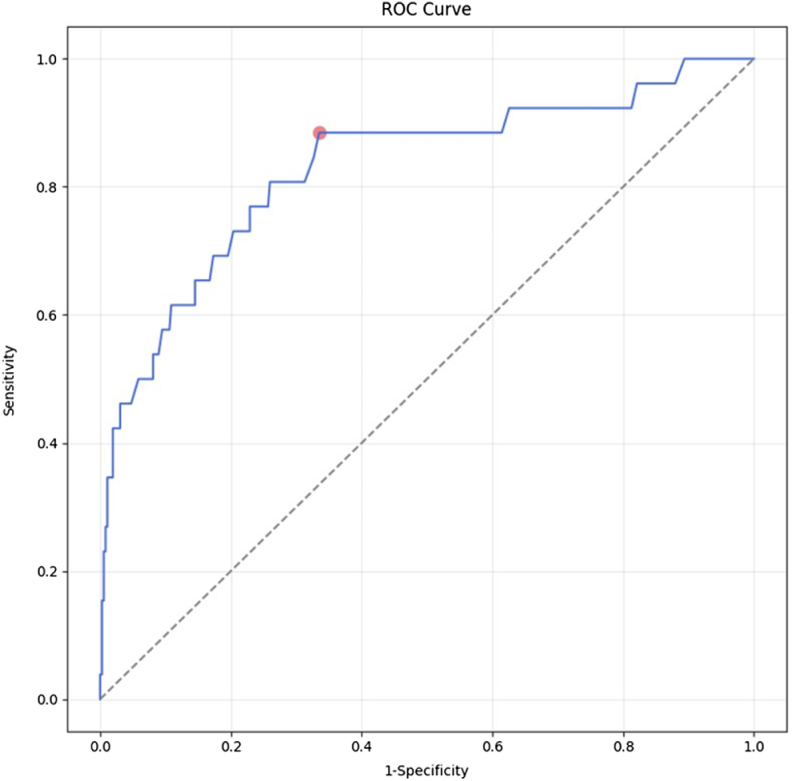
Receiver operating characteristic analysis of ICG-R15 in predicting PHLF.

ICG-R15, indocyanine green retention rate at 15 minutes; PHLF, posthepatectomy liver failure; ROC, receiver operating characteristic.

Figure 3 illustrates the ROC curve of ICG-R15 for predicting PHLF. The area under the curve (AUC) was 0.830 (95% CI: 0.735–0.922, *P* < 0.001). The optimal cut-off value for ICG-R15 was determined to be 0.069 (6.9%) with a sensitivity of 88.5% and a specificity of 66.5% (Table 2).

**Table 2:** Information of receiver operating characteristic curve in Figure 3

Variables	AUC	95% CI	Cut-off	Sensitivity	Specificity
ICG-R15	0.830	0.735–0.922	0.069	0.885	0.665

AUC, area under the curve; CI, confidence interval.

### Risk factors associated with PHLF

As shown in Table 3, in the univariate logistic regression analysis, several factors were significantly associated with PHLF, including age (OR = 1.037, 95% CI: 1.009–1.071, *P* = 0.015), surgery time (OR = 1.002, 95% CI: 1.000–1.004, *P* = 0.033), blood loss (OR = 1.001, 95% CI: 1.000–1.001, *P* = 0.013), type of occlusion (SPO vs. Pringle: OR = 0.277, 95% CI: 0.100–0.669, *P* = 0.007), ICG-R15 > 0.069 (OR = 11.329, 95% CI: 4.221–39.400, *P*<0.001), Child–Pugh grade B (OR = 5.517, 95% CI: 2.330–12.761, *P*<0.001), and undergoing>hemihepatectomy (OR = 7.514, 95% CI: 3.192–19.758, *P*<0.001).

**Table 3:** Univariate and multivariate logistic analysis of the risk factors for PHLF

Variables	*n*	Univariate analysis		Multivariate analysis	
		OR (95% CI)	*P*	OR (95% CI)	*P*
Age (years)	384	1.037 (1.009–1.071)	0.015	1.029 (0.992–1.074)	0.152
Sex					
Male	217	Reference			
Female	167	0.670 (0.279–1.511)	0.347		
BMI (kg/m2)	384	1.035 (0.924–1.157)	0.544		
Surgery time (minutes)	384	1.002 (1.000–1.004)	0.033	0.999 (0.997–1.002)	0.701
Ischemic duration (minutes)	384	0.999 (0.989–1.007)	0.868		
Blood loss (mL)	384	1.001 (1.000–1.001)	0.013	1.001 (1.000–1.001)	0.019
Type of occlusion					
Pringle	192	Reference		Reference	
SPO	192	0.277 (0.100–0.669)	0.007	0.206 (0.062–0.580)	0.005
Cirrhosis					
No	331	Reference			
Yes	53	1.147 (0.325–3.153)	0.809		
ICG-R15					
≤0.069	245	Reference		Reference	
>0.069	139	11.329 (4.221–39.400)	<0.001	8.359 (2.702–33.056)	<0.001
Child–Pugh					
Grade A	331	Reference		Reference	
Grade B	53	5.517 (2.330–12.761)	<0.001	2.823 (0.932–8.442)	0.063
Type of hepatectomy					
≤hemihepatectomy	270	Reference		Reference	
>hemihepatectomy	114	7.514 (3.192–19.758)	<0.001	6.816 (2.455–20.987)	<0.001
Lesion pathology					
Benign	162	Reference			
Malignant	222	0.995 (0.447–2.280)	0.990		

PHLF, posthepatectomy liver failure; OR, odds ratio; CI, confidence interval; BMI, body mass index; SPO, selective portal occlusion; ICG-R15, indocyanine green retention rate at 15 minutes.

In the multivariate analysis, SPO remained an independent protective factor against PHLF (OR = 0.206, 95% CI: 0.062–0.580, *P* = 0.005), while ICG-R15 > 0.069 (OR = 8.359, 95% CI: 2.702–33.056, *P* < 0.001), undergoing>hemihepatectomy (OR = 6.816, 95% CI: 2.455–20.987, *P* < 0.001), and higher intraoperative blood loss (OR = 1.001, 95% CI: 1.000–1.001, *P* = 0.019) were identified as independent risk factors.

### Subgroup analyses

**Figure 4.**
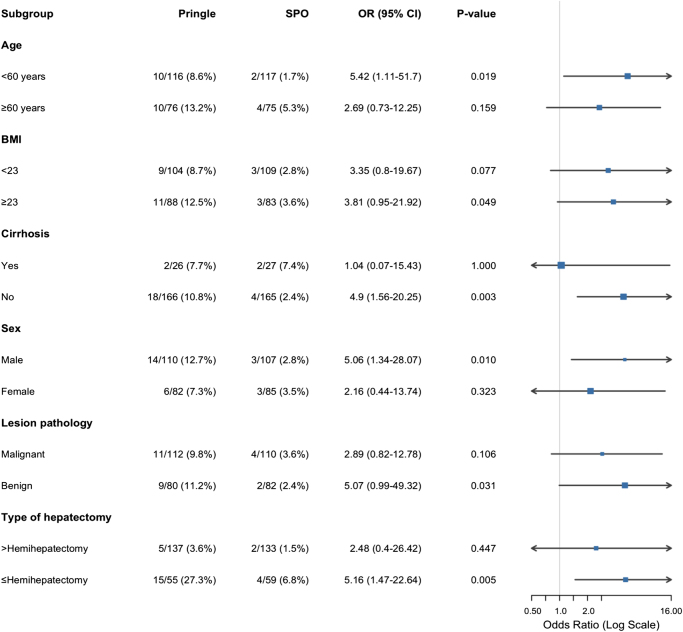
Occlusion effect on PHLF by subgroup.

PHLF, posthepatectomy liver failure; SPO, selective portal occlusion; OR, odds ratio; CI, confidence interval; BMI, body mass index.

The findings of the subgroup analyses are shown in Figure 4. It demonstrated that SPO was associated with a significantly lower incidence of PHLF compared to the Pringle maneuver in specific patient populations. The benefit of SPO was significant in patients aged<60 years (OR = 5.42, 95% CI: 1.11–51.7, *P* = 0.019), males (OR = 5.06, 95% CI: 1.34–28.07, *P* = 0.010), those with BMI≥23 (OR = 3.81, 95% CI: 0.95–21.92, *P* = 0.049), noncirrhotic patients (OR = 4.9, 95% CI: 1.56–20.25, *P* = 0.003), those with benign lesions (OR = 5.07, 95% CI: 0.99–49.32, *P* = 0.031), and those undergoing≤hemihepatectomy (OR = 5.16, 95% CI: 1.47–22.64, *P* = 0.005).

## Discussion

It is widely acknowledged that, under the background of underlying liver diseases such as cirrhosis and hepatitis, the liver remains in a state of chronic inflammation, making it more susceptible to I/R injury caused by vascular occlusion and subsequent reperfusion^[23,24]^. Therefore, minimizing such damage has been a key focus of research. Bleeding control is primarily achieved through two key approaches: reducing the hepatic inflow by controlling the portal vein and hepatic artery, like the Pringle maneuver, and minimizing venous reflux by regulating the hepatic venous outflow[12]. Based on these methods, Makuuchi *et al* and Takasaki have proposed partial liver inflow occlusion and segmental hepatic inflow occlusion, respectively, as effective strategies to actively reduce bleeding during liver resection. These methods involve occluding the blood flow only to the liver lobe or segment to be resected^[25,26]^. The advantage lies in the longer duration of occlusion compared to the Pringle maneuver, with less intraoperative liver function impairment. Additionally, these techniques preserve the blood flow to the healthy liver, reducing congestion in the portal vein and gastrointestinal tract. However, since these methods do not block the collateral vessels on the preserved liver segments, substantial bleeding may still occur at the liver transection plane, and there is considerable difficulty associated with portal vein puncture and a notable risk of portal vein thrombosis[27]. Some researchers have proposed lowering the central venous pressure (with transfusion reduced to 1 mL/kg/h) and occluding the inferior vena cava to decrease blood reflux^[28–30]^. However, these measures can negatively affect venous return from the lower limbs and kidneys, potentially leading to renal dysfunction, reduced venous return to the heart, and other hemodynamic changes. These interventions may also increase the risk of venous thrombosis and result in circulatory instability, leading to a series of adverse consequences.

In addition to blocking blood flow, some studies have explored lowering liver temperature to reduce metabolic activity, thereby decreasing intraoperative blood loss and postoperative liver dysfunction. These approaches have shown preliminary efficacy in both animal models and early clinical applications^[31,32]^. Additionally, preoperative ischemic and pharmacological preconditioning, such as the administration of antioxidants, antiinflammatory drugs, or vasodilators, has been investigated as a strategy to enhance the liver’s tolerance to I/R injury. Multiple studies have confirmed that these interventions can effectively reduce postoperative I/R injury without impairing liver regeneration^[33–36]^. Besides, the duration of vascular occlusion is directly proportional to the extent of liver damage, making the upper limit of occlusion time a critical consideration. A cumulative hepatic ischemia time of no more than 60 minutes is generally considered safe, though it has not been shown to significantly reduce mortality or morbidity rates[37]. Intermittent vascular occlusion, however, can be safely applied for a duration exceeding 120 minutes in healthy livers and may even be extended to 300 minutes if necessary. In carefully selected cirrhotic livers, a cumulative ischemia time of up to 120 minutes is deemed safe, with an upper limit of at least 200 minutes[15].

However, there has been limited exploration of vascular occlusion techniques specifically designed to reduce I/R injury. Previous studies focusing on occlusion methods that preserve partial hepatic blood flow while minimizing bleeding (selective hepatic inflow occlusion) have primarily centered on hemihepatic blood flow occlusion. This approach involves occluding the portal vein branches supplying the affected hemiliver, which is suitable for tumors confined to one side of the liver. However, this technique has significant limitations: it does not apply to tumors that cross the hemihepatic border, and it does not address bleeding from the transection plane of the preserved liver side, as vascular branches on the preserved side remain unblocked.

The liver has its unique dual blood supply. Based on this theory, we propose a novel occlusion technique involving dissecting the first hepatic hilum and selectively occluding the main portal vein while preserving hepatic arterial flow. Theoretically, the preserved hepatic artery provides an oxygen-rich blood supply to the liver tissue, thereby maximizing the reduction in I/R injury while simultaneously minimizing blood loss.

To validate the effects of this inflow occlusion technique, our surgical team conducted animal experiments using Wistar rats. The results demonstrated that, compared to the Pringle group, the SPO group exhibited higher proliferating cell nuclear antigen (PCNA) and Ki-67 labeling indexes, as well as increased technetium-99 m galactosyl human serum albumin liver uptake, indicating a more favorable environment for postoperative liver regeneration^[38–40]^. Furthermore, the SPO group showed significantly lower levels of AST, ALT, markers of inflammation, extracellular signal-regulated kinase-1/2 (ERK1/2) activation, heat shock protein 70, and interleukin-6 expression. These findings suggest reduced hepatic injury and inflammatory responses following surgery^[18,39–41]^. In a mouse hepatocarcinoma model, exploration of postoperative tumor prognosis revealed that the SPO group had lower PCNA expression and ERK1/2 activation in tumor cells, effectively inhibiting hepatocarcinoma progression by attenuating hepatic I/R injury. These results highlight the potential of the SPO technique to suppress hepatic tumor growth after hepatectomy[42].

Our team’s previous animal experiments provided robust evidence supporting the favorable effects of the SPO method on perioperative recovery and prognosis. This retrospective study was conducted to further investigate the efficacy of this occlusion technique in human body surgery and to extend and validate the findings from our prior animal research.

Our findings further validate that patients in the SPO group had a significantly lower incidence of PHLF (*P* = 0.026). Although the SPO method resulted in a somewhat prolonged surgical time during the dissection of the portal vein (median: 374 vs. 312 minutes), this difference did not reach statistical significance (*P* = 0.293). While this technique may involve slightly more intraoperative steps and technical complexity, it directly improves perioperative outcomes for patients. We believe that, on balance, the clinical benefits of SPO clearly outweigh the additional operative time and complexity. Additionally, there was no significant increase in intraoperative blood loss (median: 300 vs. 300 mL, *P* = 0.607). For a novel hepatic inflow occlusion technique in hepatectomy, it is essential to ensure that the dissection of the main portal vein is practicable, safe, and associated with an appropriate learning curve for all hepatobiliary surgeons. While this procedure is considered a fundamental skill for experienced hepatobiliary surgeons, younger surgeons may require extensive practice to achieve proficiency. Therefore, to ensure safety, this technique should ideally be implemented in large hepatobiliary centers and gradually disseminated to other institutions. In our center, a skilled surgeon typically completes the skeletonization of the first hepatic porta in no more than 60 minutes, and the time required to expose and encircle the main portal vein with a tourniquet during laparoscopic surgery can be as short as 15 minutes. In addition to this, patients in the SPO group exhibited a downward trend in postoperative BNP levels compared to those in the Pringle group (Fig. 2), particularly on POD5 (73 vs. 123 U/L, *P* = 0.035). To some extent, this suggests that the SPO method may mitigate the impact of circulatory fluctuations caused by decreased venous return on cardiac function. Further robust evidence is needed in the future to verify the potential protective effects of the SPO method on other organs throughout the body by reducing circulatory fluctuations.

Univariate and multivariate logistic regression analyses revealed that blood loss, ICG-R15 > 0.069, extended hepatectomy/trisectionectomy were independent risk factors for PHLF, while SPO was an independent protective factor. Therefore, we recommend that for patients undergoing major hepatectomy, SPO should be considered as a hepatic inflow control method. This recommendation applies after ruling out relative contraindications such as severe adhesions, anomalous vessel anatomy, or first hepatic hilum tumor invasion. Under the guidance of experienced surgeons, SPO can be safely performed following first hepatic hilum dissection, provided the extended operative time or blood loss remain within acceptable limits. This approach can effectively reduce the incidence of PHLF.

Previous literature has established that intraoperative factors like surgery time, blood loss, and extended hepatectomy can impact the incidence of PHLF^[43–48]^. In our study, these potential confounders were effectively mitigated through the application of PSM (Table 1), thereby ensuring a more precise and robust evaluation of the safety profile of the SPO. However, it is still important to acknowledge several limitations in our study. First, numerous studies have reported a positive correlation between ischemic duration and PHLF, regardless of the occlusion technique, and have established the safe upper limits for occlusion duration^[15,37]^. However, our study did not find a significant association between ischemic duration and PHLF. This may be attributed to the relatively small and homogeneous sample size. Future studies with larger cohorts are needed to provide more robust evidence. Second, as a retrospective study, it is inherently subject to selection bias, with all data being sourced from a single medical center. Third, the retrospective nature of the data collection led to instances of missing information, which may have affected the accuracy and comprehensiveness of our findings. Moreover, the absence of external validation for our dataset has somewhat weakened the reliability of our conclusions. In the future, we plan to conduct multicenter prospective randomized controlled trials to obtain more objective results and further validate the effects of the SPO technique. This will provide stronger evidence for the broader application of this method. We will also continue to report and share our findings.

## Conclusion

Under the premise of not significantly increasing blood loss and surgery time, SPO can effectively reduce the incidence of PHLF, and doesn’t increase the incidence of complications other than PHLF compared to the Pringle maneuver. For patients planned for hepatectomy, after a thorough vascular anatomical evaluation and exclusion of contraindications, SPO is advised to perform.

## Supplementary Material

## Data Availability

Data during the study are available from the corresponding author by request.

## Research registration unique identifying number (UIN)

Name of the registry: Research Registry. Unique identifying number or registration ID: researchregistry11229. Hyperlink to the registration: https://www.researchregistry.com.

## References

[1] Fan ST Lo CM Liu CL. Hepatectomy for hepatocellular carcinoma: toward zero hospital deaths. Ann Surg 1999;229:322–30. 10077043 10.1097/00000658-199903000-00004 PMC1191696

[2] Charny CK Jarnagin WR Schwartz LH. Management of 155 patients with benign liver tumours. Br J Surg 2001;88:808–13. 11412249 10.1046/j.0007-1323.2001.01771.x

[3] Lortat-Jacob JL Robert HG Henry C. Case of right segmental hepatectomy. Mem Acad Chir (Paris) 1952;78:244–51. 14947550

[4] Foster JH Berman MM. Solid liver tumors. Major Probl Clin Surg 1977;22:1–342. 839860

[5] Gayowski TJ Iwatsuki S Madariaga JR. Experience in hepatic resection for metastatic colorectal cancer: analysis of clinical and pathologic risk factors. Surgery 1994;116:703–10. 7940169 PMC2967179

[6] Nordlinger B Guiguet M Vaillant JC. Surgical resection of colorectal carcinoma metastases to the liver. A prognostic scoring system to improve case selection, based on 1568 patients. Association Française de Chirurgie. Cancer 1996;77:1254–62. 8608500

[7] Jarnagin WR Gonen M Fong Y. Improvement in perioperative outcome after hepatic resection: analysis of 1,803 consecutive cases over the past decade. Ann Surg 2002;236:397–406. 12368667 10.1097/01.SLA.0000029003.66466.B3 PMC1422593

[8] Katz SC Shia J Liau KH. Operative blood loss independently predicts recurrence and survival after resection of hepatocellular carcinoma. Ann Surg 2009;249:617–23. 19300227 10.1097/SLA.0b013e31819ed22f

[9] Huang J Tang W Hernandez-Alejandro R. Intermittent hepatic inflow occlusion during partial hepatectomy for hepatocellular carcinoma does not shorten overall survival or increase the likelihood of tumor recurrence. Medicine (Baltimore) 2014;93:e288. 25526466 10.1097/MD.0000000000000288 PMC4603114

[10] Jiang J-H Wang K-X Zhu J-Y. Comparison of hepatectomy with or without hepatic inflow occlusion in patients with hepatocellular carcinoma: a single-center experience. Minerva Med 2017;108:324–33. 28176514 10.23736/S0026-4806.17.04788-7

[11] Xia F Huang Z Ndhlovu E. The effect of the number of hepatic inflow occlusion times on the prognosis of ruptured hepatocellular carcinoma patients after hepatectomy. BMC Surg 2022;22:94. 35282826 10.1186/s12893-022-01537-8 PMC8919568

[12] Otsubo T. Control of the inflow and outflow system during liver resection. J Hepatobiliary Pancreat Sci 2012;19:15–18. 21971691 10.1007/s00534-011-0451-0

[13] Mise Y Sakamoto Y Ishizawa T. A worldwide survey of the current daily practice in liver surgery. Liver Cancer 2013;2:55–66. 24159597 10.1159/000346225 PMC3747552

[14] Hossain MA Wakabayashi H Izuishi K. The role of prostaglandins in liver ischemia-reperfusion injury. Curr Pharm Des 2006;12:2935–51. 16918423 10.2174/138161206777947678

[15] Van Riel WG van Golen RF Reiniers MJ. How much ischemia can the liver tolerate during resection? Hepatobiliary Surg Nutr 2016;5:58–71. 26904558 10.3978/j.issn.2304-3881.2015.07.05 PMC4739942

[16] Suyavaran A Thirunavukkarasu C. Preconditioning methods in the management of hepatic ischemia reperfusion- induced injury: update on molecular and future perspectives. Hepatol Res 2017;47:31–48. 26990696 10.1111/hepr.12706

[17] Zeng HJ Tang SH Qin S. Progress in the clinical diagnosis and treatment of hepatic vascular diseases. Zhonghua Gan Zang Bing Za Zhi 2020;28:977–80. 33256288 10.3760/cma.j.cn501113-20200417-00194 PMC12769897

[18] Chen YW Li CH Zhang AQ. Preserving hepatic artery flow during portal triad blood inflow occlusion reduces liver ischemia-reperfusion injury in rats. J Surg Res 2012;174:150–56. 21316704 10.1016/j.jss.2010.11.913

[19] Agha RA Mathew G Rashid R. Revised Strengthening the reporting of cohort, cross-sectional and case-control studies in surgery (STROCSS) Guideline: an update for the age of artificial intelligence. Prem J Sci 2025;10:100081.

[20] Rahbari NN Garden OJ Padbury R. Posthepatectomy liver failure: a definition and grading by the International Study Group of Liver Surgery (ISGLS). Surgery 2011;149:713–24. 21236455 10.1016/j.surg.2010.10.001

[21] Rahbari NN Garden OJ Padbury R. Post-hepatectomy haemorrhage: a definition and grading by the International Study Group of Liver Surgery (ISGLS). HPB (Oxford) 2011;13:528–35. 21762295 10.1111/j.1477-2574.2011.00319.x PMC3163274

[22] Koch M Garden OJ Padbury R. Bile leakage after hepatobiliary and pancreatic surgery: a definition and grading of severity by the International Study Group of Liver Surgery. Surgery 2011;149:680–88. 21316725 10.1016/j.surg.2010.12.002

[23] Xu Y Chen J Wang H. Perioperative and long-term outcomes of liver resection for hepatitis B virus-related hepatocellular carcinoma without versus with hepatic inflow occlusion: study protocol for a prospective randomized controlled trial. Trials 2016;17:492. 27724929 10.1186/s13063-016-1621-9 PMC5057253

[24] Isozaki H Okajima K Kobayashi M. Experimental study of liver injury after partial hepatectomy with intermittent or continuous hepatic vascular occlusion. Differences in tolerance to ischemia between normal and cirrhotic livers. Eur Surg Res 1995;27:313–22. 7589003 10.1159/000129415

[25] Makuuchi M Mori T Gunvén P. Safety of hemihepatic vascular occlusion during resection of the liver. Surg Gynecol Obstet 1987;164:155–58. 3810429

[26] Yamamoto M Katagiri S Ariizumi S. Glissonean pedicle transection method for liver surgery (with video). J Hepatobiliary Pancreat Sci 2012;19:3–8. 21938411 10.1007/s00534-011-0443-0 PMC3233661

[27] Torzilli G Makuuchi M Inoue K. No-mortality liver resection for hepatocellular carcinoma in cirrhotic and noncirrhotic patients: is there a way? A prospective analysis of our approach. Arch Surg 1999;134:984–92. 10487594 10.1001/archsurg.134.9.984

[28] Melendez JA Arslan V Fischer ME. Perioperative outcomes of major hepatic resections under low central venous pressure anesthesia: blood loss, blood transfusion, and the risk of postoperative renal dysfunction. J Am Coll Surg 1998;187:620–25. 9849736 10.1016/s1072-7515(98)00240-3

[29] Jones RM Moulton CE Hardy KJ. Central venous pressure and its effect on blood loss during liver resection. Br J Surg 1998;85:1058–60. 9717995 10.1046/j.1365-2168.1998.00795.x

[30] Otsubo T Takasaki K Yamamoto M. Bleeding during hepatectomy can be reduced by clamping the inferior vena cava below the liver. Surgery 2004;135:67–73. 14694302 10.1016/s0039-6060(03)00343-x

[31] Imakita M Yamanaka N Kuroda N. Does topical cooling alleviate ischemia/reperfusion injury during inflow occlusion in hepatectomy? Results of an experimental and clinical study. Surg Today 2000;30:795–804. 11039707 10.1007/s005950070061

[32] Kim YI Kobayashi M Nakashima K. In situ and surface liver cooling with prolonged inflow occlusion during hepatectomy in patients with chronic liver disease. Arch Surg 1994;129:620–24. 8204037 10.1001/archsurg.1994.01420300062009

[33] Feng L Wang L Rong W. Initial comparison of regional ischemic preconditioning and hemi-hepatic vascular inflow occlusion in resection of hepatocellular carcinoma. Zhonghua Zhong Liu Za Zhi 2015;37:186–89. 25975786

[34] Rodríguez A Taurà P García Domingo MI. Hepatic cytoprotective effect of ischemic and anesthetic preconditioning before liver resection when using intermittent vascular inflow occlusion: a randomized clinical trial. Surgery 2015;157:249–59. 25616941 10.1016/j.surg.2014.09.005

[35] Bahde R Spiegel H-U. Hepatic ischaemia-reperfusion injury from bench to bedside. Br J Surg 2010;97:1461–75. 20645395 10.1002/bjs.7176

[36] Zhang Y Liu M Yang Y. Dexmedetomidine exerts a protective effect on ischemia-reperfusion injury after hepatectomy: a prospective, randomized, controlled study. J Clin Anesth 2020;61:109631. 31669050 10.1016/j.jclinane.2019.109631

[37] Huguet C Gavelli A Bona S. Hepatic resection with ischemia of the liver exceeding one hour. J Am Coll Surg 1994;178:454–58. 8167881

[38] Wang P-F Li C-H Zhang A-Q. Effects of different hepatic inflow occlusion methods on liver regeneration following partial hepatectomy in rats. Zhongguo Yi Xue Ke Xue Yuan Xue Bao 2012;34:14–18. 22737713

[39] Wang PF Li CH Chen YW. Preserving hepatic artery flow during portal triad blood inflow occlusion improves remnant liver regeneration in rats after partial hepatectomy. J Surg Res 2013;181:329–36. 22878147 10.1016/j.jss.2012.07.028

[40] Kong Z Hu-J-J Ge X-L. Preserving hepatic artery flow during portal triad blood occlusion improves regeneration of the remnant liver in rats with obstructive jaundice following partial hepatectomy. Exp Ther Med 2018;16:1910–18. 30186418 10.3892/etm.2018.6402 PMC6122213

[41] Li C-H Chen Y-W Chen Y-L. Preserving low perfusion during surgical liver blood inflow control prevents hepatic microcirculatory dysfunction and irreversible hepatocyte injury in rats. Sci Rep 2015;5:14406. 26400669 10.1038/srep14406 PMC4585878

[42] Shi B Li CH Chen YW. Preserving hepatic artery flow during portal triad blood inflow occlusion reduces the outgrowth of hepatocarcinoma in mice after ischemia-reperfusion. Hepatol Res 2014;44:1224–33. 23879824 10.1111/hepr.12209

[43] Nanashima A Yamaguchi H Shibasaki S. Comparative analysis of postoperative morbidity according to type and extent of hepatectomy. Hepatogastroenterology 2005;52:844–48. 15966217

[44] Fernandez FG Ritter J Goodwin JW. Effect of steatohepatitis associated with irinotecan or oxaliplatin pretreatment on resectability of hepatic colorectal metastases. J Am Coll Surg 2005;200:845–53. 15922194 10.1016/j.jamcollsurg.2005.01.024

[45] Tomuş C Iancu C Bălă O. Liver resection for benign hepatic lesion: mortality, morbidity and risk factors for postoperative complications. Chirurgia (Bucur) 2009;104:275–80. 19601458

[46] Jin S Fu Q Wuyun G. Management of post-hepatectomy complications. World J Gastroenterol 2013;19:7983–91. 24307791 10.3748/wjg.v19.i44.7983 PMC3848145

[47] Kauffmann R Fong Y. Post-hepatectomy liver failure. Hepatobiliary Surg Nutr 2014;3:238–46. 25392835 10.3978/j.issn.2304-3881.2014.09.01 PMC4207837

[48] Shoup M Gonen M D’Angelica M. Volumetric analysis predicts hepatic dysfunction in patients undergoing major liver resection. J Gastrointest Surg 2003;7:325–30. 12654556 10.1016/s1091-255x(02)00370-0

