# Supplementary material for: Selective portal vein occlusion with hepatic artery preservation reduces posthepatectomy liver failure: a retrospective cohort study
Source: Int J Surg. 2025 Sep 24;112(1):1252–62. doi: 10.1097/JS9.0000000000003548 (PMC12825718; doi:10.1097/JS9.0000000000003548)
Supplement: Supplementary file 1 [file js9-112-1252-001.pptx]

## Slide 1
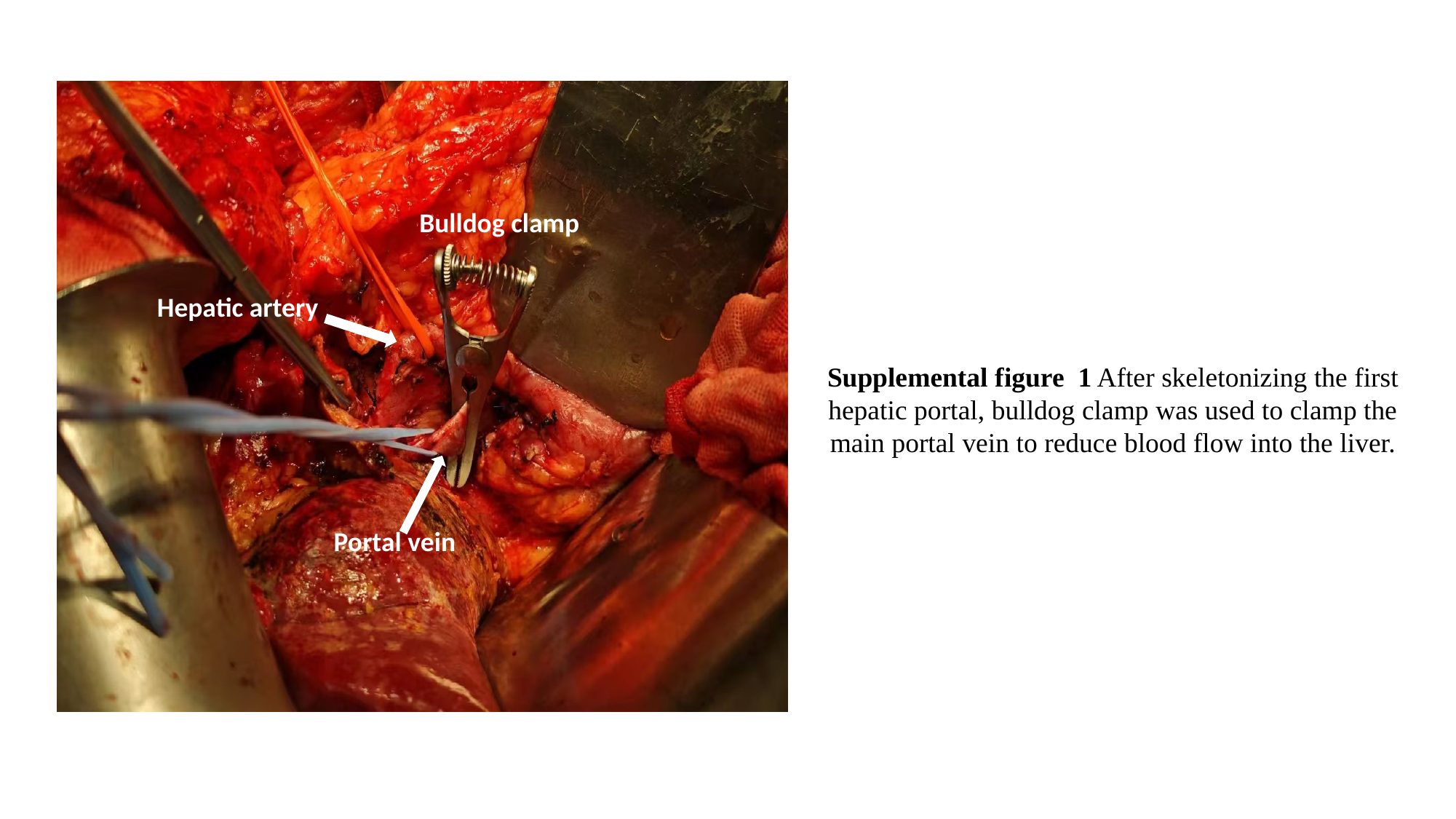

Hepatic artery
Portal vein
Bulldog clamp
Supplemental figure 1 After skeletonizing the first hepatic portal, bulldog clamp was used to clamp the main portal vein to reduce blood flow into the liver.
