# Supplementary material for: Selective portal vein occlusion with hepatic artery preservation reduces posthepatectomy liver failure: a retrospective cohort study
Source: Int J Surg. 2025 Sep 24;112(1):1252–62. doi: 10.1097/JS9.0000000000003548 (PMC12825718; doi:10.1097/JS9.0000000000003548)
Supplement: Supplementary file 2 [file js9-112-1252-002.pptx]

## Slide 1
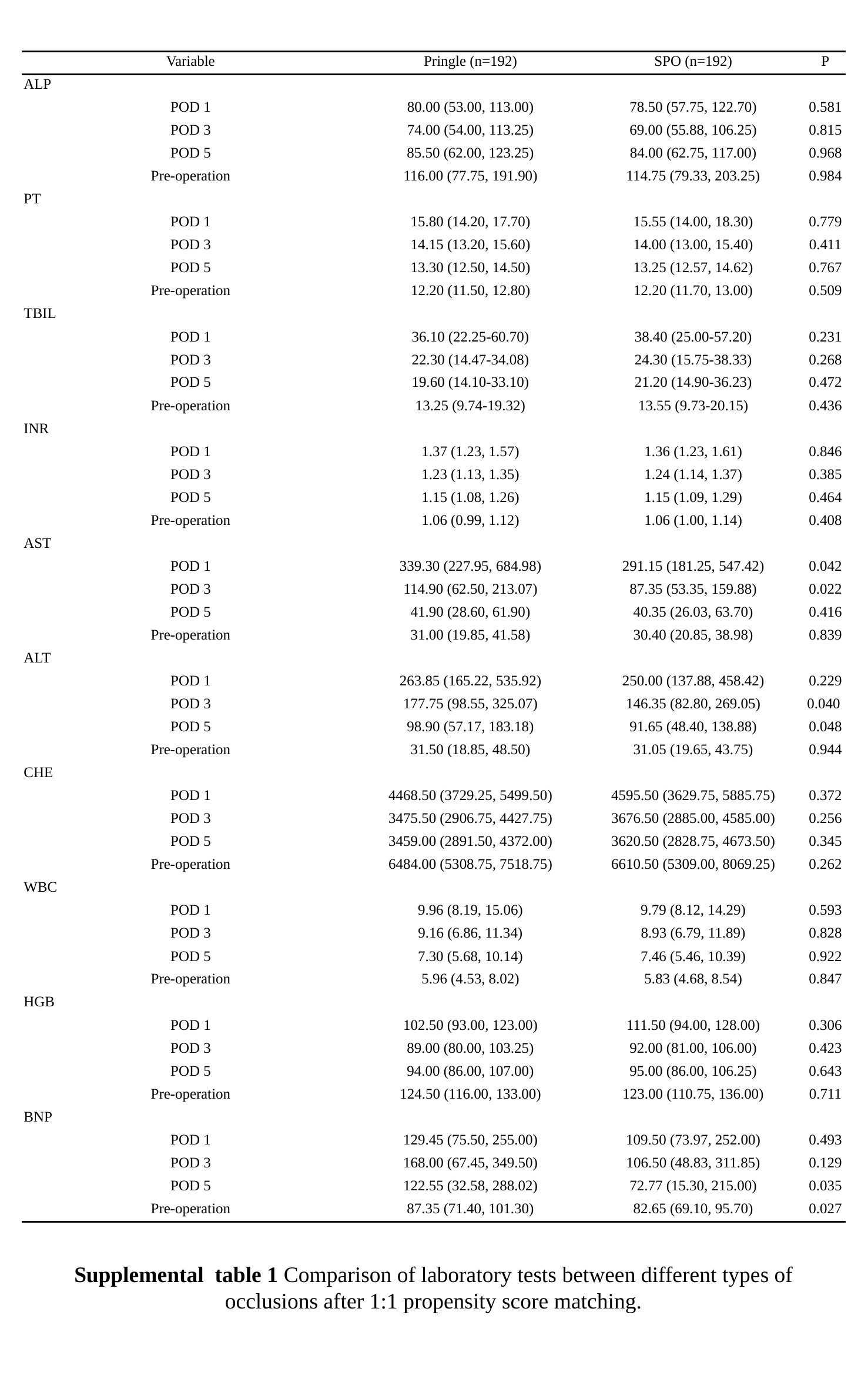

| Variable | Pringle (n=192) | SPO (n=192) | P |
| --- | --- | --- | --- |
| ALP | | | |
| POD 1 | 80.00 (53.00, 113.00) | 78.50 (57.75, 122.70) | 0.581 |
| POD 3 | 74.00 (54.00, 113.25) | 69.00 (55.88, 106.25) | 0.815 |
| POD 5 | 85.50 (62.00, 123.25) | 84.00 (62.75, 117.00) | 0.968 |
| Pre-operation | 116.00 (77.75, 191.90) | 114.75 (79.33, 203.25) | 0.984 |
| PT | | | |
| POD 1 | 15.80 (14.20, 17.70) | 15.55 (14.00, 18.30) | 0.779 |
| POD 3 | 14.15 (13.20, 15.60) | 14.00 (13.00, 15.40) | 0.411 |
| POD 5 | 13.30 (12.50, 14.50) | 13.25 (12.57, 14.62) | 0.767 |
| Pre-operation | 12.20 (11.50, 12.80) | 12.20 (11.70, 13.00) | 0.509 |
| TBIL | | | |
| POD 1 | 36.10 (22.25-60.70) | 38.40 (25.00-57.20) | 0.231 |
| POD 3 | 22.30 (14.47-34.08) | 24.30 (15.75-38.33) | 0.268 |
| POD 5 | 19.60 (14.10-33.10) | 21.20 (14.90-36.23) | 0.472 |
| Pre-operation | 13.25 (9.74-19.32) | 13.55 (9.73-20.15) | 0.436 |
| INR | | | |
| POD 1 | 1.37 (1.23, 1.57) | 1.36 (1.23, 1.61) | 0.846 |
| POD 3 | 1.23 (1.13, 1.35) | 1.24 (1.14, 1.37) | 0.385 |
| POD 5 | 1.15 (1.08, 1.26) | 1.15 (1.09, 1.29) | 0.464 |
| Pre-operation | 1.06 (0.99, 1.12) | 1.06 (1.00, 1.14) | 0.408 |
| AST | | | |
| POD 1 | 339.30 (227.95, 684.98) | 291.15 (181.25, 547.42) | 0.042 |
| POD 3 | 114.90 (62.50, 213.07) | 87.35 (53.35, 159.88) | 0.022 |
| POD 5 | 41.90 (28.60, 61.90) | 40.35 (26.03, 63.70) | 0.416 |
| Pre-operation | 31.00 (19.85, 41.58) | 30.40 (20.85, 38.98) | 0.839 |
| ALT | | | |
| POD 1 | 263.85 (165.22, 535.92) | 250.00 (137.88, 458.42) | 0.229 |
| POD 3 | 177.75 (98.55, 325.07) | 146.35 (82.80, 269.05) | 0.040 |
| POD 5 | 98.90 (57.17, 183.18) | 91.65 (48.40, 138.88) | 0.048 |
| Pre-operation | 31.50 (18.85, 48.50) | 31.05 (19.65, 43.75) | 0.944 |
| CHE | | | |
| POD 1 | 4468.50 (3729.25, 5499.50) | 4595.50 (3629.75, 5885.75) | 0.372 |
| POD 3 | 3475.50 (2906.75, 4427.75) | 3676.50 (2885.00, 4585.00) | 0.256 |
| POD 5 | 3459.00 (2891.50, 4372.00) | 3620.50 (2828.75, 4673.50) | 0.345 |
| Pre-operation | 6484.00 (5308.75, 7518.75) | 6610.50 (5309.00, 8069.25) | 0.262 |
| WBC | | | |
| POD 1 | 9.96 (8.19, 15.06) | 9.79 (8.12, 14.29) | 0.593 |
| POD 3 | 9.16 (6.86, 11.34) | 8.93 (6.79, 11.89) | 0.828 |
| POD 5 | 7.30 (5.68, 10.14) | 7.46 (5.46, 10.39) | 0.922 |
| Pre-operation | 5.96 (4.53, 8.02) | 5.83 (4.68, 8.54) | 0.847 |
| HGB | | | |
| POD 1 | 102.50 (93.00, 123.00) | 111.50 (94.00, 128.00) | 0.306 |
| POD 3 | 89.00 (80.00, 103.25) | 92.00 (81.00, 106.00) | 0.423 |
| POD 5 | 94.00 (86.00, 107.00) | 95.00 (86.00, 106.25) | 0.643 |
| Pre-operation | 124.50 (116.00, 133.00) | 123.00 (110.75, 136.00) | 0.711 |
| BNP | | | |
| POD 1 | 129.45 (75.50, 255.00) | 109.50 (73.97, 252.00) | 0.493 |
| POD 3 | 168.00 (67.45, 349.50) | 106.50 (48.83, 311.85) | 0.129 |
| POD 5 | 122.55 (32.58, 288.02) | 72.77 (15.30, 215.00) | 0.035 |
| Pre-operation | 87.35 (71.40, 101.30) | 82.65 (69.10, 95.70) | 0.027 |
Supplemental table 1 Comparison of laboratory tests between different types of occlusions after 1:1 propensity score matching.
